# Letter to the Editor regarding “Clinical benefit of high tibial osteotomy combined with the intervention of platelet-rich plasma for severe knee osteoarthritis”

**DOI:** 10.1186/s13018-023-03629-4

**Published:** 2023-03-16

**Authors:** Elena Tchetina

**Affiliations:** Immunology and Molecular Biology Laboratory, Nasonova Research Institute of Rheumatology, Moscow, Russia

I read with great interest an article by Dong et al. [1]. The authors carried out a long-term, double–blinded, randomized, placebo-controlled study aiming to reveal the therapeutic effect of platelet-rich plasma** (**PRP) versus hyaluronic acid (HA) injections or no post-treatment after open wedge high tibial osteotomy (HTO) in the end-stage patients with osteoarthritis (OA) and concluded that PRP post-treatment following HTO ended up with better surgery results in terms of functional outcomes and pain scores as well as cartilage regeneration compared with HA or no post-treatment. These results are noteworthy as they were obtained in patients with high degree of cartilage degeneration (Kellgren&Lawrence grades III and IV) although commonly HTO surgery is applied for patients with early and moderate OA with lower degree of cartilage degradation [2]. It is also worth to note that previously only three more studies with one to two years’ follow-up have been performed involving HTO associated with PRP and/or stromal vascular fraction (SVF) post-injections in patients with knee OA [3–5] and demonstrated significant improvements related to pain, cartilage healing, and clinical outcomes. However, all the above studies focused on registering structural changes only in the medial condyle and did not present records on alterations in the lateral compartment in the examined patients at the end of the follow-up. In contrast, in our preliminary 1.5-yearlong study describing clinical outcomes and synovial cytokine profiling in patients with knee OA in response to PRP or SVF post-treatments following open wedge HTO [6], we reported that SVF treatment demonstrated better outcomes according to Outerbridge and Koshino testing and produced more pronounced cartilage regeneration not only in the medial condyle but slowed down cartilage destruction in its lateral counterpart being more prominent in the femoral articular surface compared with PRP injection. At the same time, we also observed that PRP subgroup as well as SVF-treated patients demonstrated significant improvements in joint function, pain control, and cartilage regeneration compared with pre-operative state. However, PRP patients performed better in terms of functional results and pain relief (assessed by KOOS, KSS, and VAS scores) compared with the SVF subgroup. HTO functional outcomes obtained in our study are in agreement with that demonstrated by others [7, 8] whereas a combination of SVF and PRP has been shown to produce even better functional results compared with PRP alone after HTO therapy [5].

Another concern about the article by Dong et al. [1] and other abovementioned studies [3–5] is related to the absence of analyses of proinflammatory joint milieu that might affect molecular changes in the individual cells and tissues in the course of post-treatments. In view of this notion, we conducted a synovial fluid cytokine and growth factor profiling assessing 41 cytokine amounts in Multiplex Assay in the examined patients before and after both post-treatments. A significant decrease in synovial fluid concentrations of IL-6 and CXCL10 in the PRP subgroup as well as TNFα, FLT-3L, MIP-1β, RANTES, and VEGF in the SVF-treated patients might be associated with reduction of pain scores. At the same time, an augmentation of synovial growth factor concentrations such as PDGF-AB/BB in the PRP subgroup and FGF2 in patients with SVF post-treatment might be responsible for partial regeneration of the articular cartilage as it was suggested previously [9]. Multivariate Factor Analysis demonstrated that two main groups of the examined cytokines were related to each treatment procedure both prior to and one week after therapy. Moreover, the degree of connectivity estimated by STRING network analysis appeared to be stronger in both patient subgroups before treatment compared to that one week after both injections. Similar associations were observed in a recent study on synovial cytokine concentration changes during hyperacute serum treatment in human OA knee joints [10].

In conclusion, comprehensive studies of various orthobiologics associated with osteotomies including evaluation of functional outcomes at the molecular level such as analysis of proinflammatory environment and regenerative machineries should be encouraged in search for further improvement of treatment options for patients with OA.
